# Validation of mathematical model with phosphate activation effect by batch (*R*)-phenylacetylcarbinol biotransformation process utilizing *Candida tropicalis* pyruvate decarboxylase in phosphate buffer

**DOI:** 10.1038/s41598-021-91294-0

**Published:** 2021-06-03

**Authors:** Julaluk Khemacheewakul, Siraphat Taesuwan, Rojarej Nunta, Charin Techapun, Yuthana Phimolsiripol, Pornchai Rachtanapun, Kittisak Jantanasakulwong, Kritsadaporn Porninta, Sumeth Sommanee, Chatchadaporn Mahakuntha, Thanongsak Chaiyaso, Phisit Seesuriyachan, Alissara Reungsang, Ngoc Thao Ngan Trinh, Sutee Wangtueai, Sarana Rose Sommano, Noppol Leksawasdi

**Affiliations:** 1 Cluster of Agro Bio-Circular-Green Industry (Agro BCG), School of Agro-Industry, Faculty of Agro-Industry, Chiang Mai University, Chiang Mai, 50100 Thailand; 2 Faculty of Agro-Industry, Chiang Mai University, Chiang Mai, 50100 Thailand; 3 Division of Food Innovation and Business, Faculty of Agricultural Technology, Lampang Rajabhat University, Lampang, 52100 Thailand; 4 Center of Excellence in Materials Science and Technology, Faculty of Science, Chiang Mai University, Chiang Mai, 50100 Thailand; 5 Research Group for Development of Microbial Hydrogen Production Process, Khon Kaen University, Khon Kaen, 40002 Thailand; 6 Department of Biotechnology, Faculty of Technology, Khon Kaen University, Khon Kaen, 40002 Thailand; 7 Academy of Science, Royal Society of Thailand, Bangkok, 10300 Thailand; 8 Department of Food Engineering, Faculty of Food Science and Technology, Nong Lam University - Ho Chi Minh City, Linh Trung Ward, Thu Duc District, Ho Chi Minh City, 720371 Vietnam; 9 College of Maritime Studies and Management, Chiang Mai University, Samut Sakhon, 74000 Thailand; 10 Plant Bioactive Compound Laboratory, Faculty of Agriculture, Chiang Mai University, Chiang Mai, 50200 Thailand

**Keywords:** Biotechnology, Engineering, Chemical engineering, Microbiology, Applied microbiology

## Abstract

The (*R*)-phenylacetylcarbinol (PAC) batch biotransformation kinetics for partially purified *Candida tropicalis* TISTR 5350 pyruvate decarboxylase (PDC) were determined to validate a comprehensive mathematical model in 250 mL scale with 250 mM phosphate buffer/pH 7.0. PDC could convert initial 100/120 mM benzaldehyde/pyruvate substrates to the statistical significantly highest (*p* ≤ 0.05) maximum PAC concentration (95.8 ± 0.1 mM) and production rate (0.639 ± 0.001 mM min^−1^). A parameter search strategy aimed at minimizing overall residual sum of square (RSS_*T*_) based on a system of six ordinary differential equations was applied to PAC biotransformation profiles with initial benzaldehyde/pyruvate concentration of 100/120 and 30/36 mM. Ten important biotransformation kinetic parameters were then elucidated including the zeroth order activation rate constant due to phosphate buffer species (*k*_*a*_) of (9.38 ±  < 0.01) ×  10^–6^% relative PDC activity min^−1^ mM^−1^. The validation of this model to independent biotransformation kinetics with initial benzaldehyde/pyruvate concentration of 50/60 mM resulted in relatively good fitting with RSS_*T*_, mean sum of square error (MSE), and coefficient of determination (R^2^) values of 662, 17.4, and 0.9863, respectively.

## Introduction

Microbial biotransformation has been extensively used worldwide in the pharmaceutical industry followed by the food and agriculture sectors^1,2^ for almost 20 years. Revenue generated by microbial biotransformation steadily increased from USD 0.39 trillion in 2001 to USD 1.25 trillion in 2019 within the pharmaceutical industry. The food and agriculture sectors had estimated USD 1.13 trillion (2019) and USD 1.84 billion (2018) in revenue respectively from microbial biotransformation^3–5^. Although chemocatalysts can offer the relatively high catalytic activity and selectivity for some reactions^6,7^, a number of organic compounds transformation processes still rely heavily on biocatalysts to achieve the desired level of enantioselectivity^8–10^. Thus, biocatalysts including enzymes, cells organelles, and whole cells in either native or artificially constructed forms^11^ have been widely used in the production of both high-volume/low-value compounds such as ethanol^12–17^ and low-volume/high-value chemical species including (*R)*-phenylacetylcarbinol (PAC)^2,15,18–24^.

**Figure 1:**
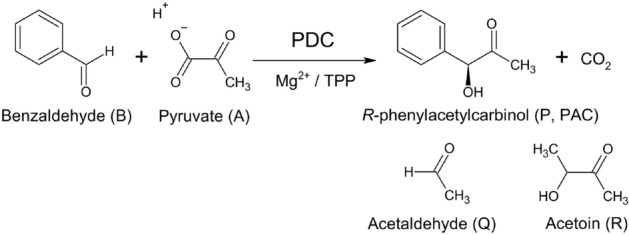
Schematic reaction mechanism of proton consuming PAC biotransformation from benzaldehyde and pyruvate substrates catalyzed by PDC with Mg^2+^ and thiamine pyrophosphate (TPP) as cofactors. The structures of acetaldehyde and acetoin by-products, resulting from direct decarboxylation of pyruvate generating free acetaldehyde and subsequent carboligation reaction of active acetaldehyde and free acetaldehyde, are also included (modified from Leksawasdi et al.^18–20^).

The biochemical production of PAC was firstly demonstrated in Germany and later commercialized for ephedrine production^25–27^. PAC could be produced through in vivo direct microbial transformation process with some strategies of benzaldehyde feeding using growing cells of yeasts, fungi, and bacteria^28–30^. This biotransformation process can be conducted in vitro by using non-viable whole cells^15,17,24^ and partially purified pyruvate decarboxylase (PDC) enzyme^18,20,28,29,31–34^. The detailed reaction mechanism of PAC biotransformation was clearly elucidated^18^ (Fig. 1). Advantages of using partially purified PDC include prevention of benzyl alcohol or PAC-diol formation. These are by-products that are often formed when PAC biotransformation is carried out in parallel with microbial cultivation process^32,35^. Moreover, the isolated enzymes could be recycled and reused during a biotransformation process^27^. The partially purified *Candida utilis* (ICI Australia) could produce 190.4 mM PAC with molar yield as high as 95.3% based on benzaldehyde^31,32,35^. The PAC concentration could generally be increased through fed-batch processes with either pyruvate (in the form of pyruvic acid) or benzaldehyde dosing protocols using 2.5 M or 20 mM of 3–morpholinopropane–1–sulfonic acid (MOPS) buffer^18–20,33^. However, the heat labile property and cost-prohibitive nature of MOPS buffer (USD 1.09/g in comparison with only USD 0.02/g for phosphate buffer) were considered major obstacles to the industrial scale application of this buffering compound^34^.

Our previous research showed that, among fifty microbial strains, *C. tropicalis* TISTR 5350 and 5306 were the best yeast strains for PAC production. The two strains yielded 70% increase in volumetric PDC carboligase activity (0.39 ± 0.06 U mL^−1^) and 58% increased PAC production (19.8 ± 3.4 mM)^21^. However, the strain TISTR 5306 was most suitable for longan extract medium^16,21–23,34^. In order to minimize cost, prevent relatively high overall losses of enzyme activity through purification process, and avoid utilization of cost ineffective MOPS buffer, Khemacheewakul et al.^34^ used whole cells *C. tropicalis* TISTR 5350 in single phase PAC production and benzaldehyde deactivation studies with various concentrations of phosphate buffer (20 mM–1.0 M). A thorough examination indicated that 1 M phosphate buffer was optimal for PAC production (28.6 ± 2.3 mM) and provided additional activation effect to PDC stability with the average PDC activation rate constant of 1.34 × 10^–2^% min^−1^. This value was higher than that observed in 20 mM phosphate buffer (1.48 × 10^–6^% min^−1^) by more than 9,050 times. Leksawasdi et al.^2^ projected that in a circulated system of recycled phosphate buffer and related co-factors utilizing whole cells of *Saccharomyces cerevisiae* and *C. tropicalis* co-culture as biocatalysts, the costing of phosphate buffer would be USD 0.751 per 1 kg of produced PAC. The similar investigation on the partially purified *C. tropicalis* TISTR 5350 PDC is thus required to evaluate relevant PAC production characteristics and assess whether the deactivation rate equation which incorporates activation effect by buffering species can interact well to the other five rate equations in a PAC biotransformation mathematical model.

The objectives of present study are to investigate the effects of initial substrates concentration pairs (benzaldehyde/pyruvate of 30/36, 50/60, and 100/120 mM) and phosphate buffer concentration levels (20, 250, 500 mM, and 1 M; pH 7.0) on PAC production, PAC formation rate, and PAC molar yields using partially purified *C. tropicalis* TISTR 5350 PDC in the 250-mL single phase batch biotransformation system. In addition, the recently improved mathematical model comprised six ordinary differential equations plus PDC-activation effect by phosphate buffering species is used to determine ten kinetic parameters based on biotransformation profiles of the 30/36 and 100/120 mM initial benzaldehyde/pyruvate concentration pairs. The model is then validated with an independent batch biotransformation system using initial substrates concentration pair of 50/60 mM followed by assessment of relevant statistical parameters.

## Materials and methods

### Microorganism

*C. tropicalis* TISTR 5350 was purchased from Thailand Institute of Scientific and Technological Research (TISTR, Pathum Thani, Thailand) before subculturing into primary stock^21^. The microbial stock was kept in 60% (v/v) glycerol solution and maintained at − 70 °C with regular assessments of cells viability by using a haemocytometer^16^. The viable cells count was 92.5 ± 1.8%^21^. *C. tropicalis* TISTR 5350 was chosen based on optimal PAC production and volumetric PDC carboligase activity in Yeast—Malt (YM) medium^16,21–23,34^.

### Cultivation of microbes and production of partially purified PDC

The cultivation of preseed and seed was done in 0.1 and 1 L yeast media as described by Khemacheewakul et al.^34^. In order to produce enough wet biomass for the production of partially purified PDC, the cultivation was carried out at 30 °C for 36 h in a 16 L stainless steel bioreactor containing 10 L inoculum medium with a composition similar to those published previously by our group^23^. The methodologies for preparation of cells pellet, glass bead pretreatment, subsequent precipitation with 50%(v/v) acetone, and production of partially purified PDC have already been stated elsewhere^22,23^.

### PAC production in phosphate buffer with various buffer and initial substrate concentration levels

The pH-controlled single-phase batch biotransformation system was done in a modified 500 mL Erlenmeyer flask containing 250 mL phosphate buffer (pH 7.0/0.1 M H_3_PO_4_) at 4 °C for 210 min with a sampling interval of 30 min^34^. Mixing was achieved by mounting the reactor on a magnetic stirrer (LMS, Model No. MGS–1001, Japan) with a magnetic bar (dia. × length of 7 × 30 mm) and speed setting of 5.0. This phosphate buffer is also known as the Gomori buffer because it contains the K_2_HPO_4_ and KH_2_PO_4_ conjugated acid–base pair^36^. A full factorial design experiment with four phosphate buffer concentration levels (20, 250, 500, and 1 M) and three pairs of initial benzaldehyde/pyruvate substrate concentration levels (30/36, 50/60 and 100/120 mM) were conducted. The molar ratio of initial pyruvate to benzaldehyde concentration was set to 1.2/ 1.0 as suggested by Leksawasdi et al.^18–20^ in order to compensate for possible pyruvate losses to by-products formation including acetaldehyde and acetoin during biotransformation process. A total of 4 × 3 = 12 groups was studied in quintuplicates. The rationale for selecting phosphate buffer at these four concentration levels utilized also by previous studies had already been elaborated in details elsewhere^34^. Partially purified PDC was added as a biocatalyst while 1 mM thiamine pyrophosphate (TPP) and 1 mM MgSO_4._7H_2_O were also included as cofactors^29,35^. Maximum PAC production level (mM), maximum PAC formation rate (mM min^−1^), and corresponding maximum PAC molar yield (the ratio of maximum PAC being produced over substrate being consumed) for each substrate pair at the maximum PAC production level were measured using high performance liquid chromatography (HPLC). The maximum PAC molar yields based on pyruvate and benzaldehyde being consumed were denoted as Y_*Pmax/A*_ and Y_*Pmax/B*_. The maximum PAC formation rate was computed by dividing maximum PAC production level at each time point with its corresponding reaction time (30, 60, 90, 120, 150, 180, and 210 min). The initial volumetric PDC carboligase activity in each experiment was maintained between 1.1 and 1.5 U carboligase mL^−1^.

### Analytical methods

The collected samples were separated into liquid buffering and enzymatic portions through centrifugation process prior to respective sample pretreatment stages as described in literatures^19,20,29,32,33,35^. PAC, benzaldehyde, benzyl alcohol, and benzoic acid concentration levels were determined by using HPLC as previously described by Rosche et al.^29^. Acetoin was analyzed by HPLC equipped with the HPX-87H column^37^. Pyruvate concentration was determined spectrophotometrically by using the enzymatic NADH + H^+^ coupled assay method with lactate dehydrogenase based on the modified method from Czok and Lamprecht^38^. Acetaldehyde concentration was determined in the same manner as the pyruvate assay with the replacement of lactate dehydrogenase with alcohol dehydrogenase based on a modified method from Bernt and Bergmeyer^39^. One unit PDC carboligase activity was defined as the rate of which 1 µmol PAC was produced from equimolar benzaldehyde and pyruvate per min in carboligase buffer at pH 6.0 and 25 °C^18^.

### Hypothesis testing

Averages and standard errors were calculated from experimental data in quintuplicates. The results obtained were statistically analyzed using SPSS for Windows 22.0 (SPSS Inc., Chicago, IL, USA) and the analysis of variance using Duncan ‘s Multiple Range Test (DMRT) at *p* ≤ 0.05^15,24,40^.

### Construction of PAC biotransformation model

The PAC biotransformation model for partially purified *C. tropicalis* TISTR 5350 PDC in this study consisted of six rate equations that described the main product (PAC) formation (1), substrates (pyruvate and benzaldehyde) consumption (2, 3), by-products (acetaldehyde and acetoin) formation (4,5) as well as deactivation of pyruvate decarboxylase with inorganic phosphate activation effect (6). Equations (1) to (5) were derived and determined based on the combined King and Altman schematic strategy as well as the initial rate studies by Leksawasdi et al.^18–20^. The original parameter constants in these rate equations were determined specifically for PAC biotransformation in 20 mM or 2.5 M MOPS buffer with partially purified *C. utilis* PDC^18–20^. Equation (6) was modified and developed by Khemacheewakul et al.^34^ for whole cells *C. tropicalis* TISTR 5350 PDC. This equation combined both the PDC deactivation effect by background species excluding buffer concentration and benzaldehyde (*k*_*d1*_, *k*_*d2*_) as well as the PDC activation effect by inorganic phosphate species (K_2_HPO_4_/KH_2_PO_4_) within the 20 mM to 1 M concentration range (*k*_*a*_). The PDC refolding effect resulting in the initial period of constant enzyme stability was also added^28^ and was denoted *t*_*lag*_ in Eq. (6). The variable *E*_*i*_ generated from this equation is crucial to the overall biotransformation system as it acts as a driving force and appears in all rate equations. The original parameter constants in this rate equation were determined from PDC deactivation kinetics in 20 mM–1 M phosphate buffer with whole cells *C. tropicalis* TISTR 5350^34^. Equation (6) has not yet been incorporated elsewhere to the PAC biotransformation model. The current study combined all six rate equations to create a PAC biotransformation model to be validated by PAC biotransformation kinetics in 250 mM phosphate buffer with partially purified *C. tropicalis* TISTR 5350 PDC.It is the first time in current study that Eq. (6) has been combined to other rate equations describing the pyruvate and benzaldehyde consumption as well as PAC and other related by-products formation to assess the overall improvement in quality of fit of mathematical model to PAC biotransformation kinetics. The complete simulation profile for each species of PAC biotransformation kinetics was constructed based on Euler–Cauchy numerical integration with a time increment of 0.01 h^18^. The description for each parameter/variable is given in the nomenclature section.

1 $$ \left. {\frac{dP}{{dt}}} \right|_{i} = V_{p} \left( {\frac{{K_{b} B_{i}^{h} }}{{1 + K_{b} B_{i}^{h} }}} \right)\left( {\frac{{A_{i} }}{{K_{ma} + A_{i} }}} \right)E_{i} $$

2 $$ \left. {\frac{dA}{{dt}}} \right|_{i} = \left. { - \frac{dP}{{dt}}} \right|_{i} \left. { - \frac{dQ}{{dt}}} \right|_{i} \left. { - 2\frac{dR}{{dt}}} \right|_{i} $$

3 $$ \left. {\frac{dB}{{dt}}} \right|_{i} = \left. { - \frac{dP}{{dt}}} \right|_{i} $$

4 $$ \left. {\frac{dQ}{{dt}}} \right|_{i} = V_{q} A_{i} E_{i} - V_{r} A_{i} Q_{i} E_{i} $$

5 $$ \left. {\frac{dR}{{dt}}} \right|_{i} = V_{r} A_{i} Q_{i} E_{i} $$

6 $$ \left. {\frac{dE}{{dt}}} \right|_{i} = \left\{ {\begin{array}{*{20}l} {0, t < t_{lag} } \hfill \\ { - \left( {k_{d1} + k_{d2} \cdot B} \right)E_{i} + k_{a} \cdot P, t \ge t_{lag} } \hfill \\ \end{array} } \right. $$

### Strategy for parameters estimation and model validation

The initial values of each parameter for Eqs. (1)–(5) were averaged from values reported in previous initial rate and simulation studies of a high-buffering-capacity biotransformation system^18,19^ using partially purified PDC from *C. utilis* in 2.5 M MOPS buffer (Table 2). The initial values for the zeroth order activation rate constant due to phosphate buffer species (*k*_*a*_) as well as the other three parameters (*k*_*d1*_, *k*_*d2*_ and *t*_*lag*_) in Eq. (6) were obtained from a recent PDC deactivation study using whole cells *C. tropicalis* TISTR 5350^34^ in 20 mM–1 M phosphate buffer. Parameter estimation and model validation were performed using the customized and well—established subroutines written in Microsoft Visual Basic for Applications (VBA) 6.3 for Microsoft Excel^12,14,18,19,34^. Specifically, the minimization of total residual sum of square (RSS_*T*_) between experimental data and simulated values from the model, and the convergence search criterion (CSC) of lesser than 1% of RSS_*T*_ were set as objective functions while the mean square error (MSE) and the coefficient of determination (R^2^) were calculated simultaneously. RSS_*T*_ for each set of biotransformation profiles is the summation of individual RSS values for product and by-products formation, substrates consumption, and enzyme deactivation profiles as shown in Eq. (7). MSE is the ratio of RSS_*T*_ and available degree of freedom (DOF) for each system. DOF is the number of experimental data points being regressed minus the total number of parameters in the mathematical model. Biotransformation profile data from the 30/36 and the 100/120 mM initial benzaldehyde/pyruvate concentration pairs were used for parameter estimation in this biotransformation model. The error estimation of each parameter was evaluated using the standard error of each time point as described by Khemacheewakul et al.^34^.

7 $$ {\text{RSS}}_{T} = {\text{RSS}}_{A} + {\text{RSS}}_{B} + {\text{RSS}}_{Q} + {\text{RSS}}_{R} + {\text{RSS}}_{E} $$

where *A* refers to pyruvate concentration, *B* refers to benzaldehyde concentration, *Q* refers to acetaldehyde concentration, *R* refers to acetoin concentration, and *E* refers to relative enzyme activity.

The estimated parameters were then validated by being implemented in Eqs. (1)–(6) and numerically integrated to simulate the biotransformation profiles of an independent data set obtained from the 50/60 mM substrate pair. The assessment of relevant statistical parameters (RSS_*T*_, MSE, R^2^) was then made to evaluate the quality of fit.

## Results

### PAC production in phosphate buffer with various buffer and initial substrate concentration levels

The current study investigated a single-phase batch biotransformation process using partially purified *C. tropicalis* TISTR 5350 PDC as a biocatalyst. The maximum PAC production levels (*P*_max_), maximum PAC formation rates (r_*P*max_), and maximum PAC molar yields with respect to each substrate (Y_*Pmax*/*B*_ and Y_*Pmax*/*A*_) under different initial substrate and phosphate buffer concentration levels are tabulated in Table 1. The 100/120 mM initial benzaldehyde/pyruvate substrate pair in 250 mM phosphate buffer showed the highest statistical significantly highest (*p* ≤ 0.05) *P*_max_ (95.8 ± 0.1 mM) and r_*P*max_ (0.639 ± 0.001 mM min^−1^) with corresponding Y_*Pmax*/*B*_ and Y_*Pmax*/*A*_ of 0.99 ± 0.01 and 0.88 ± 0.01 based on benzaldehyde and pyruvate, respectively.

**Table 1:** Concentration level effects of phosphate buffer (*Ph*_*b*_) and initial substrates (benzaldehyde (*B*) as well as pyruvate (*A*)) on maximum PAC production levels (*P*_max_), maximum PAC formation rates (r_*P*max_), as well as corresponding PAC—substrate molar yields based on benzaldehyde (Y_*Pmax*/*B*_) and pyruvate (Y_*Pmax*/*A*_) being consumed at maximum PAC production level in single—phase biotransformation systems with initial volumetric enzyme activity between 1.1 and 1.5 U carboligase mL^−1^.

(*B*/*A*) (mM)	*Ph*_*b*_ (mM)	*P*_max_ (mM)	r_*P*max_ (mM min^−1^)	Y_*P*max_ for each substrate	
				Y_*P*max/*B*_	Y_*P*max/*A*_
(30/36)	20	15.3 ± 0.26 f	0.128 ± 0.001 f	0.87 ± 0.01 c	0.62 ± 0.01 g
	250	25.8 ± 0.14 e	0.143 ± 0.001 f	**0.97 ± 0.01 ab**	**0.97 ± 0.04 ab**
	500	25.4 ± 0.28 e	0.169 ± 0.001 ef	0.95 ± 0.01 b	0.66 ± 0.01 f
	1000	15.5 ± 0.09 f	0.103 ± 0.001 g	**0.97 ± 0.01 ab**	0.81 ± 0.01 e
(50/60)	20	40.6 ± 0.21 c	0.193 ± 0.001 e	**0.99 ± 0.01 a**	**0.99 ± 0.01 a**
	250	39.2 ± 0.62 c	0.261 ± 0.001 d	**0.99 ± 0.04 a**	0.93 ± 0.01 c
	500	39.5 ± 1.14 c	0.188 ± 0.005 e	**0.96 ± 0.03 ab**	**0.99 ± 0.03 a**
	1000	35.2 ± 0.13 cd	0.168 ± 0.001 ef	**0.98 ± 0.00 ab**	0.90 ± 0.02 d
(100/120)	20	83.7 ± 0.46 b	0.465 ± 0.001 c	**0.98 ± 0.01 ab**	0.95 ± 0.01 bc
	250	**95.8 ± 0.11 a**	**0.639 ± 0.001 a**	**0.99 ± 0.01 a**	0.88 ± 0.01 d
	500	85.5 ± 0.65 b	0.407 ± 0.001 c	**0.99 ± 0.01 a**	**0.99 ± 0.01 a**
	1000	89.7 ± 0.62 b	0.598 ± 0.001 b	**0.99 ± 0.01 a**	**0.98 ± 0.01 ab**

The numbers with the same alphabet (a–g) indicated no statistical significantly difference (*p* > 0.05) for comparison between different rows of the same column within each group of initial substrates concentration pair. Bold numbers and alphabets showed the statistical highest values within each column.

The reaction time resulting in the *P*_max_ for each run could be determined by dividing *P*_max_ with r_*P*max_ whose value was the multiple of 30 min (30, 60, 90, 120, 150, 180, and 210 min). Rounding-off errors of the tabulated values might result in non-integer reaction times which should be rounded to the nearest multiple of 30 min, for instance, reaction time for PAC biotransformation with initial substrates (*B*, *A*) of (30, 36) mM in 250 mM phosphate buffer producing *P*_max_ of 25.8 mM was 25.8 mM/0.143 mM min^−1^ = 180.42 ≈ 180 min.

### PAC biotransformation model and model validation

**Figure 2:**
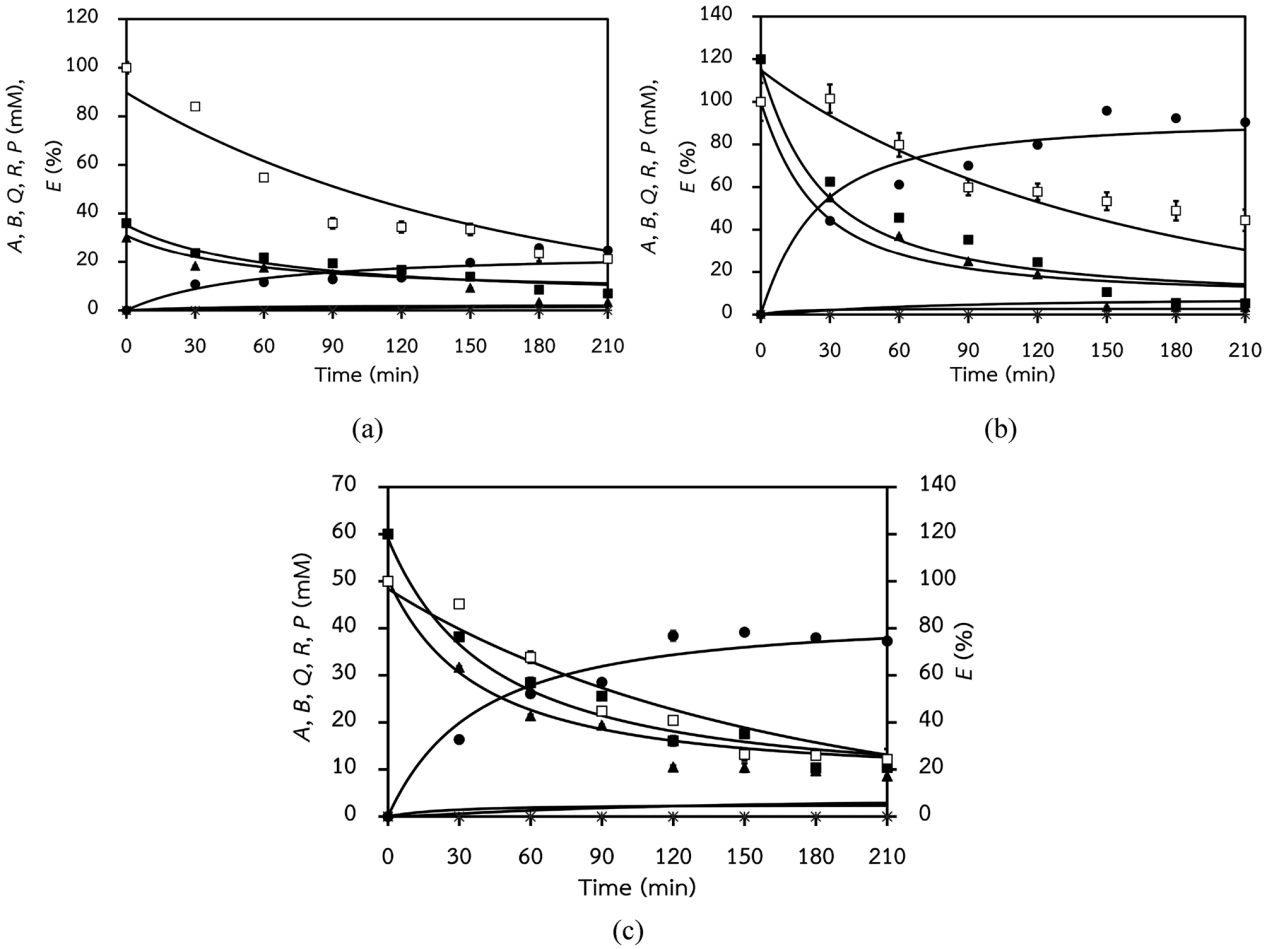
Simulated profiles of PAC (*P*, filled circle) biotransformation systems with experimental initial substrates (pyruvate—(*A*, filled square) and benzaldehyde (*B*, filled triangle)) concentration levels (mM) of (**a**): (30/36) and (**b**): (100/ 120) after RSS_*T*_ minimization as well as predicted profile for validation purpose with initial substrates concentration level of (**c**): (50/60) mM based on optimized parameter values as indicated in Table 2. The corresponding values of RSS_*T*_, MSE, and R^2^ for each profile are shown in Table 3. The error bars had already been incorporated to all experimental data sets but were found to be relatively small for most of data sets. The initial volumetric enzyme activity (*E*, white square) was 1.1–1.5 U mL^−1^. The formation of by-products such as acetaldehyde (*Q*, ×) and acetoin (*R*, +) was not detected.

Simultaneous numerical integration of rate equations Eqs. (1)–(6) was implemented to simulate a combined biotransformation kinetic profile of two benzaldehyde/pyruvate concentration pairs: 30/36 and 100/120 mM. The simulation profiles of the optimized parameters are shown in Fig. 2a,b. The initial searching values were extracted from Leksawasdi et al.^18,19^ and Khemacheewakul et al.^34^ (Table 2). Optimization of these kinetic values by a grid-search parameter estimation strategy improved fitting of the model as evident by lowering of RSS_*T*_ from 67,465 to 3,462 and MSE from 1,775 to 91.1 (19.5 times improvement). Model fitting statistics RSS_*T*_, MSE and R^2^ for individual biotransformation kinetic profiles are presented in Table 3. These were 936, 24.6 and 0.9717 for the 30/36 mM initial benzaldehyde/pyruvate concentration levels and 2,526, 66.5 and 0.9787 for the 100/120 mM levels, respectively.

**Table 2:** Initial searching values and optimized values of kinetic parameters with corresponding RSS_*T*_, MSE, and R^2^ of PAC biotransformation model for partially purified *C. tropicalis* TISTR 5350 PDC using initial substrates (pyruvate and benzaldehyde) concentration levels of (30/36) and (100/ 120) mM as well as initial volumetric enzyme activity between 1.1 and 1.5 U mL^−1^.

Kinetic parameters	Units	References	Initial searching values	Optimized values
*V*_*P*_	mM min^−1^%^−1^	(I)	1.55 × 10^–2^	(6.71 ± 0.20) × 10^–2^
*K*_*B*_	mM^1−h^	(I)	9.00 × 10^–5^	(1.01 ± 0.02) × 10^–4^
*h*	no unit	(I)	1.98	1.94 ± 0.01
*K*_*ma*_	mM	(I)	4.84	0.70 ± 0.01
*V*_*q*_	min^−1^%^−1^	(I)	6.38 × 10^–6^	(1.68 ± 0.11) × 10^–5^
*V*_*r*_	min^−1^ mM^−1^%^−1^	(I)	9.88 × 10^–7^	(6.06 ± 0.37) × 10^–6^
*k*_*d1*_	min^−1^	(II)	8.89 × 10^–3^	(5.99 ± 0.23) × 10^–3^
*k*_*d2*_	min^−1^ mM^−1^	(II)	3.30 × 10^–5^	(1.33 ± < 0.01) × 10^–5^
*k*_*a*_	% min^−1^ mM^−1^	(II)	1.34 × 10^–5^	(9.38 ± < 0.01) × 10^–6^
*t*_*lag*_	min	(II)	1.00	0.42 ± 0.37
RSS_*T*_			67,465	3,462
MSE			1,775	91.1
R^2^			0.7176	0.9772

(I): Initial rate and batch biotransformation kinetics studies by Leksawasdi et al.^18,19^ whose values were averaged and the relevant enzyme activity unit was normalized to relative percentage; (II): Benzaldehyde deactivation kinetics with inorganic phosphate buffer activation effect by Khemacheewakul et al.^34^. Full name of each parameter was given in nomenclature section.

**Table 3:** Apparent initial concentration values of chemical species (enzyme activity (*E*), pyruvate (*A*), benzaldehyde (*B*), PAC (*P*)) in the batch biotransformation processes utilized by PAC biotransformation model with corresponding individual RSS, MSE, and R^2^ for partially purified *C. tropicalis* TISTR 5350 PDC. The initial volumetric enzyme activity for each experiment was between 1.1 and 1.5 U mL^−1^ with experimental benzaldehyde and pyruvate concentration levels of (30/36), (50/60), and (100/120) mM.

Species	Units	Initial concentration of chemical species in PAC biotransformation model for each experimental *B*/*A* pair		
		30/36^a^	50/60^b^ (validation)	100/120^a^
*E*	%	89.4	96.4	114
*A*	mM	35.2	59.1	116
*B*	mM	30.9	50.4	100
*P*	mM	0.01	0.01	0.01
RSS_*T*_		936	662	2,526
MSE		24.6	17.4	66.5
R^2^		0.9717	0.9863	0.9787

^**a**^These experimental pairs were used in parameters determination process.

^**b**^The validity of parameters in PAC biotransformation model was confirmed by this experimental pair.

The model validation was done on an independent biotransformation kinetic profile with initial benzaldehyde/pyruvate concentration levels of 50/60 mM. The relatively good quality of fitting could be observed visually as shown in Fig. 2c. RSS_*T*_, MSE, and R^2^ of the fitted model were 662, 17.4, and 0.9863, respectively (Table 3).

## Discussion

The results from the effects of phosphate buffer and initial substrates concentration levels could be compared to those reported by Khemacheewakul et al.^34^ when whole cells of *C. tropicalis* TISTR 5350 was used as biocatalyst instead. Whole cells biotransformation in 1 M phosphate buffer using initial 30/40 mM benzaldehyde/pyruvate substrates concentration levels could produce *P*_*max*_, equivalent r_P*max*_, Y_*Pmax*/*B*_, and Y_*Pmax*/*A*_ of 28.6 ± 2.3 mM, 0.566 ± 0.014 mM min^−1^, 0.95 ± 0.08 and 0.71 ± 0.06, respectively. The values of *P*_max_ from both studies could not be directly compared due to different initial substrate concentration levels. Nevertheless, the results of r_*P*max_ and Y_*Pmax*/*A*_ were still comparable and statistically higher (*p* ≤ 0.05) by 12.9 ± 0.3% and 23.9 ± 2.0%, respectively for the current study which might suggest the beneficial effect of using a lower phosphate buffer concentration level (250 mM) for PAC biotransformation system with partially purified PDC. In fact, *P*_max_ (15.5 ± 0.1 mM) and r_*P*max_ (0.103 ± 0.001 mM min^−1^) of partially purified *C. tropicalis* TISTR 5350 PDC were significantly affected (*p* ≤ 0.05) by 1 M phosphate buffer in comparison with the whole cells counterpart (45.8 ± 4.4% and 81.8 ± 0.5% lowered, respectively) when subjected to similar initial substrates concentration level as shown in Table 1. As PDC is an intracellular enzyme, the application of this enzyme in the form of whole cells may protect the enzyme against high phosphate buffer concentrations through the mechanism of mass transfer limitation due to diffusion hindrance. However, catalytic rates may be compromised ^41^. This was also in agreement with Rosche et al.^32^ who revealed that 600 mM phosphate buffer had greater level of inhibitory effect to partially purified PDC from *Rhizopus javanicus* NRRL 13,161 than MOPS buffer at a similar concentration level resulting in lower PAC concentration being produced. MOPS buffer was then chosen as buffer of choice for subsequent studies^18–20,28,33^. In fact, partially purified *C. utilis* PDC in 2.5 M MOPS buffer also expressed the highest r_*P*max_ when initial benzaldehyde/pyruvate concentration level was 100/120 mM^18^. Evidently, Y_*Pmax*/*B*_ was not affected by phosphate buffer concentration at 250 mM and 1 M as both molar yields were in the vicinity of unity and not statistically significant different (*p* > 0.05) from one another.

Several authors had discussed advantages and disadvantages of using phosphate species as reaction buffers for PDC^2,25,32,34,42^. Juni et al.^42^ pointed out that high phosphate concentration might help prevent proteolytic enzymes from deactivating PDC, thereby enhancing the PDC stability while strengthening association of important prosthetic cofactors such as TPP with the enzyme. Such protective effect from a high phosphate buffer concentration observed in this study may also be applicable to PDC from *R. javanicus* NRRL 13161, because it possesses a relatively high PAC formation rate^32^. *R. javanicus* was formerly ranked as a potential candidate for the best PAC producer. However, the possibility of large-scale enzyme production from this filamentous fungal strain was eventually abandoned due to unsolvable proteolytic enzyme problems (unpublished result). Van Urk et al.^25^ discovered allosteric inhibition of phosphate species on *S. cerevisiae* PDC in a greater extent than that of *C. utilis*. Using phosphate buffer for PAC biotransformation with *C. tropicalis* TISTR 5350 PDC may be advantageous when conducted in an optimal concentration for each enzyme form (i.e., 1 M for whole cells PDC^34^ and 250 mM for partially purified PDC (this study)). Phosphate buffer also offers cost-saving advantage. Leksawasdi et al.^17^ proposed that utilization of phosphate buffer in a 1 kg PAC production system would maintain the cost ratio of reaction buffer to total production at 25.9%, which is much lower than a cost ratio of 94.9% for MOPS buffer^34^.

Evidently, the developed PAC biotransformation model provided good fitting within the investigated range of 30/36 to 100/120 mM initial benzaldehyde/pyruvate concentration levels for partially purified *C. tropicalis* TISTR 5350 PDC. Comparison of all ten kinetic parameters determined in the current study to those with comparable normalized units in the literature is given in Table 4. *k*_*a*_ was included to PAC biotransformation for the first time in current study. Khemacheewakul et al.^34^ reported *k*_*a*_ value of (13.4 ± 8.6) × 10^–6^% min^−1^ mM^−1^ which was not statistical significantly different (*p* > 0.05) from current study suggesting similar activation effect between 1 M phosphate buffer on whole cells *C. tropicalis* TISTR 5350 and 250 mM phosphate buffer on partially purified *C. tropicalis* TISTR 5350 PDC. The inclusion of *k*_*a*_*.P* term in Eq. (6) allowed the effect of buffering species to be separated out from the original enzyme deactivation model which contained only *k*_*d1*_, *k*_*d2*_, and *t*_*lag*_. The absence of *k*_*a*_*.P* term in previous studies would result in averaging out of buffer species activation effect to aforementioned three parameters with relatively less quality of fit as shown in Table 4. The activation effect due to buffering species and corresponding buffer concentration should therefore be included as separated term in enzyme deactivation rate equation for overall improvement of PAC biotransformation model’s predictive capability. The developed PAC biotransformation model could also be applied in a wider range to assess quantitatively the effect of buffering species on PAC biotransformation under different conditions by comparing the respective *k*_*a*_ value in each system. The statistical significantly highest (*p* ≤ 0.05) overall rate constant for the formation of PAC (*V*_*p*_) by using partially purified *C. tropicalis* TISTR 5350 PDC indicated that PDC from this strain produces PAC faster than that from *C. utilis* by 4–5 times^18,19^. The improvement in value of *V*_*p*_ was thus in agreement with finding in previous section where 250 mM phosphate buffer was utilized. Further comparison was made between intrinsic binding constant (*K*_*b*_) and Hill coefficient (*h*) for benzaldehyde whose values for both partially purified PDC from *C. utilis* and *C. tropicalis* was not statistical significantly different (*p* > 0.05) with validated range of mean between (0.80–1.01) × 10^–4^ mM^1−h^ and 1.77–2.18, respectively^18,19^. It was possible that partially purified PDC from both strains exhibited similar allosteric and sigmoidal behaviors toward benzaldehyde. In addition, *h* value of ~ 2 suggests that PDC might operate in a dimeric subunit form during PAC biotransformation^18–20^. The lower affinity constant value for pyruvate (*K*_*ma*_) in the current study implies a greater level of binding between partially purified PDC from *C. tropicalis* TISTR 5350 and pyruvate than the *C. utilis* counterpart by 9–11 times. In fact, the observably higher overall rate constants for the formation of both acetaldehyde (*V*_*q*_) and acetoin (*V*_*r*_) in the current study may be the result of increased affinity toward pyruvate of this decarboxylating enzyme^18–20,25,31^. Partially purified PDC from *C. tropicalis* TISTR 5350 was more prone to deactivation effect by benzaldehyde than the *C. utilis* counterpart as indicated by a much higher first-order benzaldehyde deactivation coefficient (*k*_*d2*_) and shorter lag time for *C. tropicalis* PDC. However, the use of phosphate buffer could negate a portion this deactivating effect by providing additional activation effect. The multiplication between zeroth-order activation rate constant due to phosphate buffer (*k*_*a*_) with PDC stabilizing effect of 250 mM phosphate buffer resulted in an activation rate of (2.34 ±  < 0.02) × 10^–3^% min^−1^. This rate was lower/higher than the activation rates when whole cell PDC in 1 M (1.34 × 10^–2^% min^−1^) and 20 mM (1.48 × 10^–6^% min^−1^) phosphate buffer were used^34^.

**Table 4:** Comparison of normalized kinetic parameters from literatures and current study.

Kinetic para-meters	Units	Leksawasdi et al.^18 a^		Leksawasdi et al. ^19 b^		Current study^c^	
*V*_*P*_	mM min^−1^%^−1^	(1.30 ± 0.02) × 10^–2^	c	(1.56 ± 0.03) × 10^–2^	b	**(6.71 ± 0.20) × 10**^**–2**^	**a**
*K*_*B*_	mM^1−h^	**(1.00 ± < 0.01) × 10**^**–4**^	**a**	**(0.80 ± < 0.01) × 10**^**–4**^	**a**	**(1.01 ± 0.02) × 10**^**–4**^	**a**
*h*	no unit	**2.18 ± 0.58**	**a**	**1.77 ± 0.47**	**a**	**1.94 ± 0.01**	**a**
*K*_*ma*_	mM	**7.91 ± 8.21**^**d**^	**a,b**	**6.33 ± 0.10**	**a**	0.70 ± 0.01	b
*V*_*q*_	min^−1^%^−1^	(0.61 ± < 0.01) × 10^–5^	b	(0.03 ± < 0.01) × 10^–5^	c	**(1.68 ± 0.11) × 10**^**–5**^	**a**
*V*_*r*_	min^−1^ mM^−1^%^−1^	(0.98 ± < 0.01) × 10^–6^	b	(0.01 ± < 0.01) × 10^–6^	c	**(6.06 ± 0.37) × 10**^**–6**^	**a**
*k*_*d1*_	min^−1^	(0.04 ± < 0.01) × 10^–3^	c	(0.05 ± < 0.01) × 10^–3^	b	**(5.99 ± 0.23) × 10**^**–3**^	**a**
*k*_*d2*_	min^−1^ mM^−1^	(0.33 ± < 0.01) × 10^–5^	c	(0.40 ± < 0.01) × 10^–5^	b	**(1.33 ± < 0.01) × 10**^**–5**^	**a**
*k*_*a*_^e^	% min^−1^ mM^−1^	N/a^e^		N/a^e^		(9.38 ± < 0.01) × 10^–6 e^	
*t*_*lag*_	min	**314 ± < 1**	**a**	252 ± < 1	b	0.42 ± 0.37	c
Prediction capability		Interpolation		Extrapolation		Interpolation	
Validated initial (*B*, *A*) in mM		(100, 120)		(400, 600)		(50, 60)	
RSS_*T*_^f^		1610		11,000		**662**	
MSE^f^		28.2		282		**17.4**	
R^2 f.^		** ~ 0.99**		~ 0.97		** ~ 0.99**	

Values with different capital alphabets (a—c) in the same row indicated statistically significant difference (*p* ≤ 0.05).

Statistical parameters with the highest statistically significant (*p* ≤ 0.05) or best values were bolded.

^a^2.5 M MOPS with (*B*/*A*) of (50/60), (100/120), (150/180) mM using initial volumetric PDC activity of 1.1–3.4 U mL^−1^.

^b^2.5 M MOPS with (*B*/*A*) of (400/600) mM using initial volumetric PDC activity of 8.4 U mL^−1^.

^c^1 M phosphate with (*B*/*A*) of (30/36), (50/60), (100/120) mM using initial volumetric PDC activity of 1.1–1.5 U mL^−1^.

^d^Large error of *K*_*ma*_ was reported by this literature.

^e^*k*_*a*_ was only included in current study, Khemacheewakul et al.^34^ reported *k*_*a*_ value of (13.4 ± 8.6) × 10^–6^% min^−1^ mM^−1^ which was not statistical significantly different (*p* > 0.05) from current study.

^f^These statistical parameters were normalized across previous studies of Leksawasdi et al.^18,19^ and current study so that RSS_*T*_, MSE, and R^2^ were comparable.

The molarity balancing of PAC formation was examined by observing PAC molar yield based on each substrate. The ranges for Y_*Pmax*/*B*_ were 0.85–0.97, 0.89–0.99, and 0.93–0.99 for the initial benzaldehyde and pyruvate concentration pairs of 30/36, 50/60, and 100/120 mM, respectively, indicating the closing molarity balance. On the contrary, corresponding ranges of of Y_*Pmax*/*A*_ of 0.80–0.97, 0.81–0.93, and 0.78–0.89 were not uniform among the tested concentration pairs. Similar phenomenon was also observed in other PAC biotransformation systems^18–20,29,31,32,34,35^. The formation of relatively volatile by-products such as acetaldehyde from pyruvate generally resulted in lower ranges of Y_*Pmax*/*A*_ in comparison with Y_*Pmax*/*B*_. The absence of acetaldehyde concentration in the reaction buffer and some losses in pyruvate molarity balance confirmed the volatility nature of this compound^18–20^. Benzyl alcohol, benzoic acid and acetoin were also not detected in the current study. Benzyl alcohol and its derivatives are often formed from side reactions of either alcohol dehydrogenase or other oxidoreductases when growing yeast cells were used in a PAC biotransformation process, resulting in a loss of up to 30–40% benzaldehyde^26,43^.

## Conclusions

The partially purified *C. tropicalis* TISTR 5350 PDC with initial volumetric enzyme activity between 1.1 and 1.5 U mL^−1^ produced PAC at the optimized levels (95.8 ± 0.1 mM and 0.639 ± 0.001 mM min^−1^) in 250 mM phosphate buffer. The improved mathematical model fitted well to the PAC biotransformation kinetics of two initial benzaldehyde and pyruvate concentration levels at 30/36 and 100/120 mM. The independent prediction of 50/60 mM benzaldehyde/pyruvate profile validated the interpolation ability of the developed model with corresponding RSS_*T*_, MSE, and R^2^ of 662, 17.4, and 0.9863, respectively. Such mathematical model will be useful for further optimization of a more complex biotransformation process, for instance, development of feeding strategies in fed batch or continuous systems.

## Data Availability

The datasets generated during and/or analysed during the current study are available from the corresponding author on reasonable request.

## List of symbols

*A*: Pyruvate concentration (mM)
*B*: Benzaldehyde concentration (mM)
CSC: Convergence search criterion (no unit)
*E*: Relative enzyme activity (%)
*h*: Hill coefficient for benzaldehyde (no unit)
*i*: Iteration loop identifier for numerical integration
*k*_*a*_: Zeroth order activation rate constant due to phosphate buffer (% relative enzyme activity min^−1^ mM^−1^ of phosphate buffer)
*k*_*d1*_: First order reaction time deactivation constant (min^−1^)
*k*_*d2*_: First order benzaldehyde deactivation coefficient (mM^−1^ min^−1^)
*K*_*b*_: Intrinsic binding constant for benzaldehyde (mM^1−h^)
*K*_*ma*_: Affinity constant for pyruvate (mM)
MSE: Mean square error (no unit)
N/a: Not available
*P*: *R*-phenylacetylcarbinol concentration (mM)
PAC: *R*-phenylacetylcarbinol
*Ph*_***b***_: Phosphate buffer concentration (mM)
PDC: Pyruvate decarboxylase enzyme
*Q*: Acetaldehyde concentration (mM)
r: Formation rate(mM min^−1^)
*R*: Acetoin concentration (mM)
R^2^: Coefficient of determination (no unit)
RSS: Residual sum of squares (no unit)
*t*: Time (min)
*T*: Total substrates and products species within the reaction buffer
*t*_*lag*_: Lag time due to enzyme refolding effect describing period of constant stability (min)
U: Carboligase activity of PDC (unit)
*V*_*p*_: Overall rate constant for the formation of PAC (mM min^−1^%^−1^)
*V*_*q*_: Overall rate constant for the formation of acetaldehyde (min^−1^%^−1^)
*V*_*r*_: Overall rate constant for the formation of acetoin (min^−1^ mM^−1^%^−1^)
Y: Molar yield of *R*-phenylacetylcarbinol on each substrate (no unit)
